# Impact of the Coronavirus on Providing Oral Health Care in the Netherlands

**DOI:** 10.1016/j.identj.2021.09.003

**Published:** 2021-09-21

**Authors:** Ilona F. Persoon, Catherine M.C. Volgenant, Monique H. van der Veen, Niek J.M. Opdam, David J. Manton, Josef J.M. Bruers

**Affiliations:** a Department of Preventive Dentistry, Academic Centre for Dentistry Amsterdam (ACTA), University of Amsterdam and Vrije Universiteit Amsterdam, Amsterdam, the Netherlands; b Department of Oral Hygiene, InHolland University of Applied Sciences, Amsterdam, the Netherlands; c Department of Dentistry, Radboud UMC, Nijmegen, The Netherlands; d Cariology and Paediatric Dentistry, University Medical Centre Groningen (UMCG), Groningen, The Netherlands; e Department of Oral Public Health, Academic Centre for Dentistry Amsterdam (ACTA), University of Amsterdam and Vrije Universiteit Amsterdam, Amsterdam, the Netherlands; f Royal Dutch Dental Association (KNMT), Utrecht, The Netherlands

**Keywords:** SARS-CoV-2, COVID-19, Dental infection control, Dental care, Dental practice management, Dentists

## Abstract

**Objective:**

Transmission of SARS-CoV-2 during oral health care is potentially increased compared to regular social activities. Specific amendments to the Dutch national infection control guidelines were promulgated. This study aimed to map the impact of the coronavirus pandemic on providing oral health care during the first wave of the coronavirus pandemic in 2020 in the Netherlands.

**Methods:**

A cross-sectional web-based survey was sent via email to a representative sample of dental hygienists and dentists in the Netherlands.

**Results:**

Of the 1700 oral health care practitioners approached, 440 (25.9%) responded to the survey. Patient access to oral health care was severely restricted during the lockdown in the spring of 2020. A total of 1.6% of the oral health care practitioners had laboratory-confirmed COVID-19 during the study period, although this is likely to be an underrepresentation due to limited access to testing at that time. Over half of the participants perceived an increased risk of virus transmission during aerosol-generating treatments in their practices. A large majority (65.0%–87.1%) of the oral health care practitioners followed the COVID-19-specific amendments to the national infection control guidelines. Compared to the pre-pandemic period, additional personal protective equipment and protocols were applied. Factors related with compliance with the additional recommendations were age, employment status, and occupation.

**Conclusions:**

The pandemic had a profound impact on both the accessibility and practice of oral health care. This survey study found that most Dutch oral health care practitioners paid extra attention to hygiene and infection control. Also, a low number of COVID-19 infections detected amongst Dutch oral health care practitioners was reported in the Netherlands. These overall outcomes suggest that safe oral health care can be provided when following the current infection control recommendations.

## Introduction

Severe acute respiratory syndrome coronavirus type 2 (SARS-CoV-2) was first reported in China in late 2019.^1^ This virus can cause coronavirus disease-19 (COVID-19). On 20 January 2020, human-to-human transmission of this virus was demonstrated for the first time and on 11 March 2020, the World Health Organisation (WHO) declared the COVID-19 outbreak a pandemic. In mid-March, many countries, including the Netherlands, went into lockdown, also ceasing all regular oral health care activities.

Symptoms of COVID-19 are diverse and usually appear 2 to 14 days after infection with SARS-CoV-2. Severe illness develops in 19% of cases.^1^ These patients require ventilation in intensive care units and some cases have a fatal outcome, mainly amongst people older than 68 years who have comorbidity.^1^ Not everyone infected with the virus experiences symptoms, experiences symptoms immediately, or recognises their symptoms immediately.^2^ These asymptomatic and presymptomatic individuals can be infectious.^3^^,^^4^ Asymptomatic, presymptomatic, and symptomatic people can carry similar numbers of virus particles.^5^ Therefore, containing the spread of the virus is complicated by dispersal by asymptomatic and presymptomatic individuals.

The SARS-CoV-2 virus is transmissible via 3 main routes^6^^,^^7^: (1) direct transmission occurs through droplets (coughing, sneezing, and medical procedures) within a distance of 1 to 2 metres^6^; (2) airborne transmission occurs via inhalation of aerosols containing very small droplets and particles containing the virus^8^^,^^9^; and (3) indirect transmission may occur through contaminated surfaces and fomites.^10^ This requires the transfer of sufficient infectious material to mucous membranes, most often via the hands.

Increased transmission in oral health care settings cannot be ruled out.^11^ Viral particles can be detected in both saliva^12^ and gingival crevicular fluid (GCF).^13^ Moreover, the spread of virus particles may be enhanced by aerosol-generating procedures using highspeed air-rotors, ultrasonic scaling equipment, or slow-speed handpieces.^14^ An increased prevalence of SARS-CoV-2 infection has been reported amongst health care workers.^15^ The prevalence of COVID-19 amongst oral health care practitioners is 1.9% for dentists and 0.8% for dental assistants in France and 0.9% amongst US dentists.^16^^,^^17^

In oral health care, all kinds of additional measures are recommended to prevent infection.^18^ Worldwide, different considerations and choices are made in this regard.^19^^,^^20^ In the Netherlands, contemporaneous with the national lockdown, the professional associations for oral health care recommended suspension of regular care and only provision of emergency care to patients without symptoms of COVID-19. Subsequently, COVID-19-specific amendments to the national infection control guideline were drafted (see Table 1)^21^ after which regular oral health care was resumed Mid-April 2020. The current survey aims to map the impact of the coronavirus pandemic on providing oral health care during the first wave of the corona pandemic in 2020 in the Netherlands.

**Table 1:** Summary of the SARS-CoV-2 amendments on infection control in oral health care in the Netherlands.^21^

*Domain*	*Description*
Triage	Question patients for symptoms of COVID-19 or possible risk factors
General public measures	Social distancing (1.5 metres), frequent hand hygiene, no handshaking, stay at home and test in case of COVID-19 symptoms
Personal protective equipment	Surgical face mask II (EN14683) with face shield or IIR with glasses, instead of minimum surgical face mask II with glasses, nonsterile examination gloves (similar as before pandemic)
Appointments and reception	No patient companions, limit waiting time, minimise contact with objects in the waiting room, avoid exchanging paper (invoice, referral letter) and pay digitally or by card
Patient treatment	Ventilation of treatment room, removal of superfluous objects, rinse with hydrogen peroxide before aerosol-generating procedures, use rubber dam, use high-volume evacuator

## Methods

This cross-sectional survey study was conducted as a web-based survey sent to a sample of dental hygienists and dentists in the Netherlands. The survey used was developed in collaboration with an international working group (COVIDental Collaboration Group), in which 36 research groups participated worldwide.^22^^,^^23^ The global survey was developed using a modified Delphi method and pretested by 12 dental professionals.^23^ For the Dutch survey, the questions were translated from English into Dutch, after which this translation was verified by an independent colleague with experience in both oral health care and international research. Several questions in the survey were adapted or added in accordance with the Dutch context. In total, 35 questions in 4 domains were included in the survey: (1) demographic data (age, sex, area of residence and work, employment status, occupation); (2) health and COVID-19 (symptoms of COVID-19); (3) personal protective equipment (PPE) and working conditions; and (4) self-perceived additional risk of contracting COVID-19.^23^ The study protocol for this study was approved by the ACTA Internal Ethics Review Board (internal registration number 2020covid02). Informed consent was obtained from all participants.

The research population comprised random samples of 1200 dentists and 500 dental hygienists obtained from the Royal Dutch Dental Association (KNMT; regarding dentists) and from the Dutch Society for Dental Hygienists (NVM; regarding dental hygienists), which are the representative organisations of both professions. In order to guarantee the anonymity of the participants, the web survey was conducted by an independent research agency (KBA Nijmegen, the Netherlands) using the open-source survey tool LimeSurvey (LimeSurvey GmbH, Hamburg, Germany). Dental hygienists and dentists were invited by email on 8 July 2020, which included a personalised link to the survey. Three reminders were sent, on July 15 and August 5 and 12, after which data collection was closed on 28 August 2020. This anonymised database was made available to the researchers.

The data were analysed with Statistical Package for Social Sciences (IBM SPSS Statistics for Windows, version 24.0; IBM Corp.). The usual statistical measures for distribution and dispersion were used to describe the data. Coherence of sex, age, occupation, employment status, and perceived infection risk with following the recommendations on several domains in full (yes/no) as dependent variables was consecutively assessed using univariate logistic regression. Thereafter, multivariate logistic regression analysis was performed. Odds ratios with corresponding 95% confidence intervals were calculated.

## Results

### Response and representativeness

Of the 1700 oral health care practitioners approached, 440 (25.9%) responded to the survey: 304 of 1200 (25.3%) dentists and 136 of 500 (27.2%) dental hygienists. Respondents were representative of the Dutch population of dentists and dental hygienists with regard to sex and geographic distribution (*p* > .05). Oral health care practitioners aged 49 years or younger were slightly underrepresented amongst the respondents compared to nonrespondents (58.2% vs 67.6%), *χ*^2^ (1) = 12.797; *p* < .001. Respondents who indicated that they were no longer actively treating patients were excluded from the analysis, resulting in a final study group of 433 respondents: 298 dentists and 135 dental hygienists. Table 2 provides demographic characteristics of the respondents.

**Table 2:** Personal and occupational characteristics of respondents.

	Dentists	Dental hygienists	Total
Sex*			
- Male	50.7%	1.5%	35.5%
- Female	49.3%	98.5%	64.5%
Age (on 1/1/2020)†			
- 29 years or younger	4.3%	17.7%	8.4%
- 30-39 years	19.1%	22.8%	20.2%
- 40-49 years	24.7%	30.1%	26.4%
- 50-59 years	25.3%	23.5%	24.8%
- 60 years or older	26.6%	5.9%	20.2%
Average (SD)	49.3 (11.6)	42.0 (11.1)	47.1 (11.9)
Employment status‡			
- Practice owner	59.5%	30.1%	50.5%
- Employee/self-employed	40.5%	69.9%	49.5%
Patient treatment §			
- Active	98.0%	99.3%	98.4%
- Not active	2.0%	0.7%	1.6%
n	304	136	440

⁎ *χ*^2^ (1) = 99.341, *p* < .001.

† *χ*^2^ (4) = 41.835, *p* < .001. *F* (1) = 38.059, *p* < 0.001.

‡ *χ*^2^ (1) = 32.473, *p* < .001.

§ *χ*^2^ (1) = 0.920, *p* = .337.

### COVID-19 symptoms

During the first wave, 19.2% of respondents had experienced potential symptoms of COVID-19. These were mostly fatigue (10.2%), coughing (9.9%), nasal congestion (9.0%), sore throat (8.8%), and headache (8.0%). Compared to dentists, proportionally more dental hygienists experienced these symptoms: 16.4% vs 25.2%, *χ*^2^ (1) = 4.583; *p* = .032.

Of the 83 respondents who experienced symptoms, 54 (65.1%) indicated that they had been tested for the presence of SARS-CoV-2 and 7 (13.0%) tested positive. Based on all respondents (n = 433), the prevalence of laboratory-confirmed SARS-CoV-2 virus infection was 1.6% (95% confidence interval [CI], 0.7-3.4). No respondents with symptoms of COVID-19 had been admitted to hospital.

### Provision of oral health care

Respondents estimated the oral health care treatments performed from March until June 2020 as compared to 2019. Few respondents indicated that they treated similar numbers of patients in March and April (1.7% and 0.7%, respectively). This percentage increased to 26.7% in May and further to 70.7% in June (Supporting Material Table S1). The number of patients treated did not significantly differ with regard to age, sex, or employment status of the oral health care practitioner. However, compared to dentists, dental hygienists performed relatively fewer patient treatments in all months.

### Personal protective equipment

A large proportion of respondents increased their use of face and head protection with a higher degree of protection (Supporting Material Table S2). The amended guideline on PPE was followed “completely” or “more than completely” by 81.5% of the respondents (Table 3). When the guideline was followed “more than completely,” this included the use of respirators, sterile gloves, aprons, and surgical caps.

**Table 3:** Compliance with the SARS-CoV-2 amendments to the national infection control guideline.

	Personal protective equipment	Appointment planning	Patient reception	Patient treatment
Does not follow guideline	0.2%	0.5%	0.5%	3.6%
Follows guideline partly	18.3%	34.5%	16.5%	9.3%
Follows guideline	33.2%	15.5%	27.3%	1.7%
Follows guideline plus	48.3%	49.5%	55.7%	85.4%
n	422	412	411	410

### Appointment planning and patient reception

After resuming regular oral health care mid-April, almost all respondents took additional measures with regard to appointment planning and patient reception (Supporting Material Table S3). Additional recommendations regarding appointment planning were followed completely by 65.0% and partially by 35.0% of the respondents in most situations (Table 3). Additional recommendations regarding patient reception were followed fully by 83.0% and partially by 17.0% of the respondents.

### Patient treatment

Oral health care practitioners took many additional measures to prevent transmission during patient treatment (Supporting Material Table S4). Additional recommendations were followed fully by 87.1%, partially by 9.3%, and not at all by 3.6% of the respondents (Table 3).

### Perceived infection risk

Of the respondents, 22.8% perceived an increased risk of SARS-CoV-2 virus infection when treating patients to be likely for both patients and oral health care practitioners. In addition, 38.6% considered only oral health care practitioners to be at risk and 0.5% considered only patients to be at risk. Furthermore, 38.1% of the respondents estimated that neither patients nor oral health care practitioners were likely to become infected with SARS-CoV-2 during treatment. The perceived infection risks were not related to sex, age, employment status, or occupation.

### Compliance with the guidelines

Being older, a dentist, or a practice owner or perceiving an increased infection risk for the patient contributed to more compliance with the guidelines (univariate analyses; Table 4). In the multivariate analysis, only practice owners, compared to non–practice owners, followed the guidelines on PPE in full more often (Table 4). The recommendations for appointment planning and patient reception were followed in full more often by dentists than dental hygienists. Recommendations on patient reception were followed more often in full by non–practice owners compared to practice owners. Finally, older oral health care practitioners followed the recommendations in full more often on patient treatment.

**Table 4:** Compliance with the SARS-CoV-2 amendments to the national infection control guideline in relation to characteristics of oral health care practitioners.

Odds ratio univariate regression* (95% CI)	Personal protective equipment	Appointment planning	Patient reception	Patient treatment
Female	0.73 (0.44-1.23)	0.76 (0.50-1.18)	0.95 (0.55-1.63)	1.48 (0.82-2.65)
Age	1.04 (1.02-1.06)†	1.02 (1.00-1.04)†	0.99 (0.97-1.01)	1.03 (1.00-1.05)†
Dental hygienist	0.46 (0.28-0.75)†	0.40 (0.26-0.62)†	0.52 (0.31-0.89)†	1.26 (0.66-2.41)
Practice owner	3.55 (2.10-6.01)†	0.94 (0.62-1.40)	0.54 (0.32-0.92)†	1.13 (0.63-2.01)
Perceived infection risk for OHCP	0.98 (0.49-2.01)	1.14 (0.75-1.73)	1.17 (0.69-2.00)	1.20 (0.66-2.16)
Perceived infection risk for patient	0.40 (0.18-0.85)†	0.76 (0.47-1.22)	1.18 (0.63-2.35)	0.90 (0.46-1.77)

Odds ratio multivariate regression* (95% CI)	Personal protective equipment	Appointment planning	Patient reception	Patient treatment
Female	1.70 (0.82-3.51)	1.35 (0.78-2.32)	1.22 (0.60-2.49)	1.90 (0.91-3.95)
Age	1.02 (1.00-1.05)	1.02 (0.99-1.04)	0.99 (0.97-1.02)	1.04 (1.01-1.07)†
Dental hygienist	0.54 (0.28-1.05)	0.35 (0.20-0.59)†	0.36 (0.18-0.72)†	1.18 (0.54-2.60)
Practice owner	2.97 (1.60-5.51)†	0.65 (0.41-1.04)	0.45 (0.24-0.82)†	0.92 (0.47-1.79)
Perceived infection risk for OHCP	1.12 (0.60-2.09)	1.13 (0.70-1.84)	1.00 (0.54-1.83)	1.36 (0.69-2.68)
Perceived infection risk for patient	0.80 (0.40-1.59)	0.83 (0.48-1.45)	1.21 (0.58-2.52)	0.83 (0.38-1.82)

Nagelkerke *R*^2^	0.122	0.076	0.061	0.048

⁎ Logistic regression was performed on respondents not or partly following guideline (0) versus respondents completely or more than completely following guideline (1).

† *p* < .05. OHCP, oral health care practitioner.

## Discussion

### SARS-CoV-2 contamination amongst oral health care practitioners

In the Netherlands, the global SARS-CoV-2 pandemic has had clear effects on both the accessibility and the provision of oral health care. The pandemic led to an increased compliance with infection control measures in oral health care. Increased compliance during the SARS-CoV-2 pandemic has also been reported in hospitals, resulting in fewer hospital-acquired infections such as methicillin-resistant *Staphylococcus aureus*.^24^^,^^25^ Furthermore, more than 60% of the study participants believed that oral health care practitioners were at an increased risk for contracting COVID-19 while treating patients.^26^^,^^27^ In Italy, a larger proportion of dentists (70%-85%) who were concerned about contracting COVID-19 was reported.

Only 65% of the respondents with COVID-19-related complaints were tested due to limited testing availability at the time of the survey; of these, 13% tested positive for the virus. Therefore, the limited confirmed prevalence of the SARS-CoV-2 virus in this study group (1.6%; 95% CI, 0.7-3.4) cannot be extrapolated to oral health care practitioners in general. However, this low prevalence falls within the range of what has been reported elsewhere, such as 0.9% confirmed infections in Dutch health care workers,^28^ 0.9% confirmed infections in oral health care practitioners in the US,^17^ and 0.9% in Swiss oral health care practitioners.^29^

### Infection control measurements in the dental office

Little evidence is available on the efficacy of additional measures to prevent SARS-CoV-2 infections. Appropriate scientific evidence and knowledge regarding oral health care practitioners contribute to implementation of and compliance with new measures. There is little scientific evidence to recommend mouth rinses to reduce the oral viral load of SARS-CoV-2; therefore, further (preferably) clinical studies are needed.^30^, ^31^, ^32^, ^33^

It is important to carefully apply cleaning and disinfection in the treatment room, as this is an effective measure to prevent transmission of the virus.^34^, ^35^, ^36^, ^37^ Also, due to the long survival of the virus on skin, the appropriate application of hand hygiene and glove use is of great importance.^38^

Regarding PPE, major changes can be seen in the use of mouth masks and eye protection. Many oral health care practitioners started using IIR or FFP2 masks in the March to June period. FFP2 masks are respiratory protection devices and offer protection of a higher order than II/IIR surgical mouth masks. In the Netherlands, FFP2 masks are recommended for, amongst other things, aerosol generating procedures in COVID-19 patients.^39^ This diverse picture regarding the use of masks by oral health care practitioners is reflected worldwide, as there has been widely varying advice in this area.^19^^,^^20^

A mere 0.7% of respondents reported that they were able to treat as many patients during the lockdown period in spring 2020 as in pre-pandemic times. Similar dramatic decreases in productivity were observed in other countries.^26^^,^^28^^,^^40^^,^^41^ By June, 68.5% of respondents were performing similar numbers of treatments as before the pandemic.

### Compliance with measurements and perceived risks

Factors related to compliance with the recommendations were age, employment status, and occupation. An Australian research group reported that dentists followed the infection prevention guidelines more often than dental hygienists,^42^ although a US study found no differences.^43^ The difference in type of patient treatment, degree of aerosol production, and number of patients per day between dental hygienists and dentists may explain the observed differences in the Netherlands. Dental hygienists indicated that they performed fewer patient treatments during the study period, purportedly due to not being involved frequently in providing acute oral health care. Other factors involved in compliance are also acknowledged in general health care, with younger doctors and women being more fastidious with hygiene practices.^44^ However, a Hong Kong study reported that older nurses had a higher level of compliance with preventive measures.^45^ Respondents from that study with a chronic health conditions were also more inclined to follow the guidelines.

Over half of the respondents perceived an increased risk of virus transmission during aerosol-based treatments in oral health care practices. This concern is also reported in studies from Italy and Jordan.^25^^,^^26^^,^^46^ The virus has been detected in GCF and saliva,^12^^,^^13^ although only indirect indications of an actual increased risk of transmission are available in the literature.^47^, ^48^, ^49^ This perceived increased risk of infection contributes to psychological distress and physical complaints amongst oral health care practitioners.^41^^,^^50^, ^51^, ^52^, ^53^

### Limitations and recommendations

Due to the restrictions imposed because of the pandemic, it was only possible to perform an online survey study to approach the participants. This could lead to potential survey error in which the true situation may deviate from the reported situation.^54^ The analysis of the representation of the respondents did not show large deviations from the general population of Dutch oral health care practitioners. . Concurrently, the significance of these results should be interpreted with caution. The effect and necessity of the various additional infection control measures taken in oral health care practices are still largely unknown. More research into the various infection control measures will contribute to safe and effective oral health care for both patients and oral health care practitioners.

## Conclusions

The COVID-19 lockdown during the spring of 2020 in the Netherlands had a major negative short-term effect on patient access to oral health care. This survey study found that most Dutch oral health care practitioners paid extra attention to hygiene and infection control. Also, a low number of COVID-19 infections detected amongst oral health care practitioners in the Netherlands was reported. These overall outcomes suggest that safe oral health care can be provided when following the current infection control recommendations.

## Appendix Supplementary materials

Image, application 1
