# Supplementary material for: Impact of the Coronavirus on Providing Oral Health Care in the Netherlands
Source: Int Dent J. 2021 Sep 21;72(4):545–51. doi: 10.1016/j.identj.2021.09.003 (PMC8452527; doi:10.1016/j.identj.2021.09.003)
Supplement: Supplementary file 1 [file mmc1.pdf]

Supporting material

Table S1 Percentage of estimated patient treatments March to June 2020, differentiated for dental hygienists and dentists [#1]

	March	April	May	June
No patient treatment	19.9%	37.0%	5.0%	4.5%
Fewer patient treatments	78.4%	62.3%	68.3%	24.8%
As many patient treatments	1.7%	0.7%	26.7%	70.7%
n	403	403	403	403
Z based on T(x+1)-T(x) [#2]		-6.19	13.12	12.09
p-value		< 0.01	< 0.01	< 0.01

	March		April		May		June	
	yes	no	yes	no	yes	no	yes	no
Dental hygienist (n = 123)	68.3%	31.7%	45.5%	54.5%	90.0%	10.0%	91.9%	8.1%
Dentist (n = 280)	85.4%	14.6%	70.7%	29.3%	97.1%	2.9%	97.1%	2.9%
Total (n = 403) [#3]	80.1%	19.9%	63.0%	37.0%	95.0%	5.0%	95.5%	4.5%
X^2(df=2)	16.599		31.962		18.785		7.392	
p-value	< 0.001		< 0.001		< 0.001		0.025	

#1 The data relate to the estimated percentages of patient treatments per month, expressed as the proportion of the number of patients who visited the practice in 2019. When two or more answers were given, the answer with the highest proportion was used. The results were recoded: 1-75% of the normal patient numbers were recoded as 'fewer patient treatments' and 76-100% and > 100% of the normal patient numbers as 'as many patient treatments'.

#2 Wilcoxon test

#3 Not all participants answered all questions.

Table S2 Personal protective equipment used by respondents in aerosol generating procedures, before the pandemic and during the pandemic

Before pandemic	During pandemic				
**Facemask**	type unknown	type II(R)	FFP1, FFP2 or FFP3	no reply	total
type unknown	5.5%	9.2%	3.1%	1.2%	19.0%
type II(R)	0.5%	56.2%	13.0%	4.7%	74.4%
FFP1, FFP2 or FFP3		0.7%	3.6%	0.7%	5.0%
no reply	0.2%	0.9%		0.5%	1.7%
Total	6.2%	67.0%	19.7%	7.1%	100%
$X^2 = 239.934$ / df = 9 / $p < 0.001$					

**Safety glasses / face shield**	none	glasses	shield	glasses / shield	total
none	3.3%	2.1%	8.1%	1.9%	15.4%
glasses	1.7%	20.6%	19.2%	39.1%	80.6%
shield		0.2%	2.1%		2.4%
glasses / shield	0.9%			0.7%	1.7%
Total	5.9%	23.0%	29.4%	41.7%	100%
$X^2 = 123.823$ / df = 9 / $p < 0.001$					

**Disposable gloves**	no gloves	non-sterile	sterile	sterile / non-sterile	total
no gloves	0.7%	2.6%			3.3%
non-sterile	6.6%	78.0%	0.2%	0.5%	85.3%
sterile	0.7%		4.5%		5.2%
sterile / non-sterile	1.0%			5.2%	6.2%
Total	9.0%	80.6%	4.7%	5.7%	100%
$X^2 = 678.418$ / df = 9 / $p < 0.001$					

**Disposable apron** #1	no apron	non-sterile	sterile	sterile / non-sterile	total
no apron	88.4%	6.5%	1.0%	0.7%	96.6%
non-sterile	0.2%	0.7%			1.0%
sterile	0.7%		1.2%		1.9%
sterile / non-sterile	0.2%			0.2%	0.5%
Total	89.7%	7.2%	2.2%	0.9%	100%
$X^2 = 217.666$ / df = 9 / $p < 0.001$					

**Surgical cap**	no	yes	total
no	78.0%	17.5%	95.5%
yes	0.7%	3.8%	4.5%
Total	78.7%	21.3%	100%
$X^2 = 48.890$ / df = 1 / $p < 0.001$			

n = 422

same protection since pandemic (yellow)

more protection since pandemic (blue)

less protection since pandemic (green)

#1 Water-repellent and other types of disposable aprons are combined

Table S3 Additional precautions taken by respondents with regard to appointment planning and reception at the practice when treating non-COVID-19 suspected patients

**Appointment planning**	
No additional precautions	0.5%
Additional precautions	99.5%
- triage on COVID-19 symptoms or risk factors	98.8%
- no patient companions	85.4%
- fewer appointments, so that the waiting room is not crowded	73.3%
- postponing appointments for elderly/vulnerable patients	68.2%
- deferring non-urgent appointments, such as check-ups	41.7%
**Reception at practice**	
No additional precautions	0.5%
Additional precautions	99.5%
- remove magazines/books from waiting room	98.3%
- patient hand hygiene	97.8%
- space of at least 1.5 meters between patients	93.4%
- physical barrier at reception/maintain 1.5-meter distance	90.3%
- more frequent ventilation of waiting room	51.6%
- keep coats, bags and other items outside treatment area	39.6%
- gloves for patient	1.2%
- mask for patient	0.7%

n = 411-412

Strongly recommended measure (grey shading)

Optional measure (peach shading)

Table S4 Additional precautions taken by respondents with regard to patient contact when treating non-COVID-19 suspected patients

No additional precautions	0.2%
Additional precautions	99.8%
- cleaning/disinfection of handles/doorhandles several times a day	95.1%
- cleaning/disinfection of push buttons/chairs several times a day	88.3%
- apply preoperative mouth rinse by patient[#]	86.6%
- increased ventilation of the treatment room during aerosol generating procedures	56.3%
- apply more/larger suction during treatment	42.7%
- use less aerosol generating procedures	41.2%
- use of rubber dam more frequently	36.3%
- education/training of team on COVID-19	21.7%
- ventilation of the treatment room for at least 10 minutes after each patient	18.1%
- application of digital care (e-Health)	7.6%
- working in a room with negative pressure	2.7%
- measuring body temperature of patients	15.1%
- measuring body temperature of all employees	10.2%
n = 409 - 410	

Strongly recommended measure (grey)
Optional measure (orange)
Measure not mentioned in guidelines (blue)

# The 355 oral health care practitioners who reported using preoperative mouth rinses used hydrogen peroxide (1.00%) in 97.5% of cases and chlorhexidine (0.12%-0.20%) in 2.5% of cases.
